# Temperature-Based Design Method for Concrete Cone Failure Under Fire Conditions

**DOI:** 10.3390/ma19143071

**Published:** 2026-07-16

**Authors:** Nicolas Pinoteau, Kresimir Nincevic, Kenton McBride, Killian Regnier, Antoine Labbé, Roberto Piccinin

**Affiliations:** 1 Centre Scientifique et Technique du Bâtiment (CSTB), 77447 Champs-sur-Marne, France; 2 Hilti Entwicklungsgesellschaft mbH, 86916 Kaufering, Germany; 3 Hilti North America, Plano, TX 75024, USA; kenton.mcbride@hilti.com; 4 Hilti Corporation, 9494 Schaan, Liechtenstein

**Keywords:** concrete cone failure, fire resistance, anchors, Eurocode 2, temperature-based design

## Abstract

This paper introduces a temperature-based design method for determining the tensile concrete cone capacity of anchors subjected to fire exposure. Current prescriptive approaches, most notably Eurocode 2, rely solely on embedment depth and fire duration for fire exposure from one or more directions on one concrete face with and without edge effects. The purpose of this work is to develop a temperature-based design method for predicting tensile concrete cone failure of anchors under fire conditions. Through an experimental program, this study demonstrates that anchor capacity can be conservatively described by temperature associated with embedment depth. A parametric analysis on the influence of concrete strength, anchor group effect, edge effect, concrete cracking, anchor type and heating orientation on different concrete faces defines the scope of application of the design method. The proposed method incorporates measured temperature profiles, offering a more precise alternative to the existing design method in Eurocode 2 and enabling flexible design for different structural configurations and fire scenarios. Optimized design is enabled in comparison to the existing prescriptive method (EN 1992-4) exclusively based on embedment depth and fire duration. Research findings demonstrate that anchor capacity correlates strongly with the temperature at the effective embedment depth under varying conditions.

## 1. Introduction

Anchorage systems are commonly used to transfer loads from structural and nonstructural steel elements to concrete members. Multiple anchor technologies exist today, broadly categorized into cast-in anchors (installed before concrete casting) and post-installed anchors (installed into cured concrete using mechanical and/or adhesive systems). Cast-in anchors are widely used in new constructions and offer efficient transfer mechanisms and direct tie-in to cast-in reinforcement. Post-installed anchors offer flexibility for renovation, retrofitting, and in new constructions where drilling into cured concrete is favorable [1]. Their use in both new and existing structures is linked to modern construction practices demanding adaptability and speed on construction sites.

Product qualification is generally addressed by the Construction Product Regulation (CPR) through European Assessment Documents in Europe and the International Code Council Evaluation Service (ICC-ES) through Acceptance Criteria in the United States. Product assessment is linked to design methods described in the European Organization for Technical Assessment (EOTA), Technical Reports or in EN 1992-4 [2] for fastening systems in Europe and in ACI 318-25 [3] for fastening systems in the USA. Additionally, the fib MC 2020 [4] provides international guidance for designing concrete structures including anchoring and reinforcing bars. Both qualification and design cover critical failure modes including steel, pullout, concrete cone, combined bond and concrete failure, concrete splitting, and concrete blow-out for static, quasi-static, and seismic loading. The regulatory framework for fire loading conditions, however, is less developed. Only bonded adhesive anchors and reinforcing bars benefit from dedicated qualification and design guidance for fire conditions through EAD 330087-01-0601 [5], EOTA TR020 [6], ICC-ES AC308 [7], and ACI 355.5 [8]. Steel failure for reinforcing bars is covered by EN 1992-1-2 [9] and ACI 216.1 [10]. In recent years, bond failure has been assessed as a priority due to the vulnerability of polymer adhesives to high temperatures [11,12]. Previous research allowed researchers to propose a small-scale characterization of the bond systems and a design method that was validated through full-scale structural fire testing [13,14,15]. Mechanical post-installed and cast-in anchors still lack such comprehensive fire-specific design provisions despite their widespread use. The fire design of concrete-related failure modes is covered in the informative Annex D of EN 1992-4 [2] for fires rating up to 120 min for ISO 834-1 conventional heating [16] based on the background research from Reick in 2001 [17]. Unlike the design for bond and steel failure under fire, the design method for concrete cone under fire is prescriptive and not based on temperature, thus limiting the scope of application and preventing optimized design. Since 2001, other authors have studied concrete cone failure experimentally and numerically at high temperatures [18,19,20,21,22] as described in Section 2 and quantified the residual tensile and shear capacity after fire exposure [22,23,24].

The goal of this paper is to establish a design method for tensile concrete cone failure under fire based on temperature. A major challenge in developing such a method is the shortage of consistent fire test data. Experimental results available in the literature are sparse, difficult to compare, and often limited to specific embedment depths or anchor types. The experimental research program presented in this paper interrogates use of the temperature at the embedment depth as the governing parameter of concrete cone failure under fire conditions. To address issues of inconsistency and generate clearer trends, the present research introduces the use of radiant heating panels to produce controlled and repeatable thermal fields. This technique enables a systematic parametric analysis of key variables including crack presence, heating orientation, group effects, concrete strength, and fastener technology. By combining these experimental insights with previous studies on conventional fire curves, this work establishes a foundation for a temperature-based design method for predicting concrete cone failure under fire. The paper is structured as follows:

1. A review of previous research that led to existing prescriptive fire design methods.
2. The proposal of a new performance-based design methodology relying explicitly on temperature as the governing variable.
3. A parametric investigation examining the influence of cracks, heating configuration, group action, concrete strength, and anchor type.

A reduction factor associated with the temperature at the embedment depth is used to describe the effect. The proposed design method fills a design gap by offering a performance-based approach in addition to the existing prescriptive method solely based on embedment depth and fire duration (with limited applicability to complex thermal configurations (multi-sided heating, different fire scenarios)), which often leads to overly conservative design predictions.

## 2. Literature Review

### 2.1. Scientific Background

**Figure 1:**
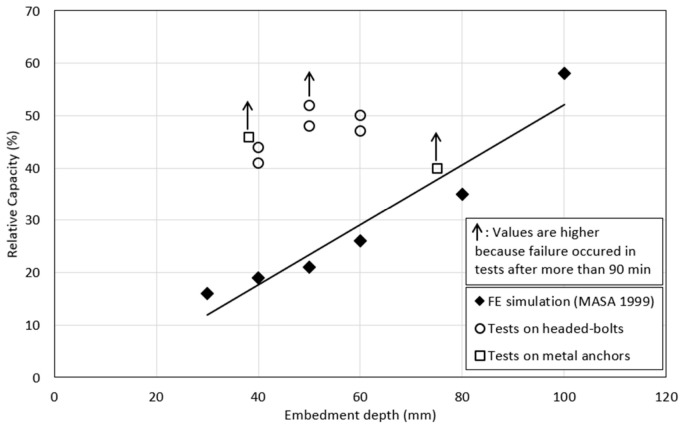
Variation of tensile capacity versus embedment depth determined by Reick [17] through tests and simulation.

In 2001, Reick [17] proposed a descriptive model on concrete cone failure derived from physical fire tests and Finite Element Modeling using MASA [20]. This investigation included headed and expansion anchors with depths of 30 to 100 mm [17]. Using physical and simulated test data at failures approximately within the range of 90 and 120 min, a relationship based on a reduction coefficient equal to the embedment length (h_ef_) normalized to 200 mm was established (Figure 1). This coefficient was implemented as part of the prescriptive fire design method in Annex D of EN 1992-4 [2]. Because the only parameter that influences the decay of capacity under fire conditions is the embedment depth (h_ef_), there is no dependency on product technology. This model produces a substantial decay of capacity during the first minutes of fire exposure. As an example, for a typical embedment depth (h_ef_ = 100 mm), the reduction coefficient (h_ef_/200) is equal to 0.5 in the first few minutes of an ISO fire event.

**Figure 2:**
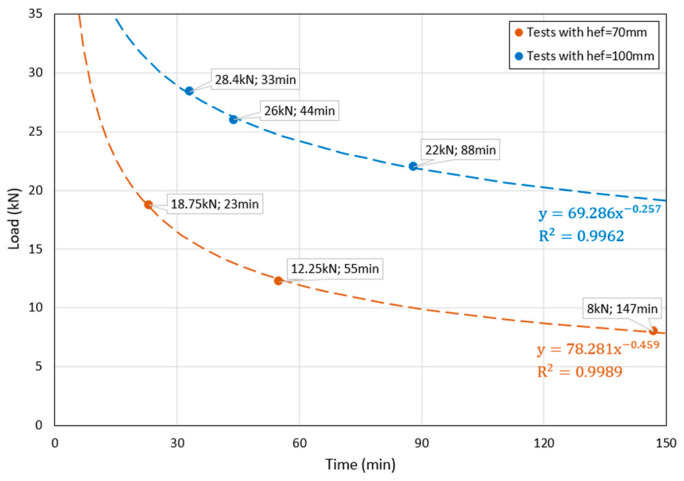
Variation of load capacity versus time determined by Lakhani et al. [25] from tests on expansion anchors.

In 2023, Lakhani and Hofmann [25] developed decay curves from fire tests performed on expansion anchors at embedment depths of 70 and 100 mm. In addition to confirming that concrete cone failures can occur during fire exposure (in addition to steel and pull-out failure), the study confirmed observations by Reick that a rapid decay occurs during the early stages of exposure (Figure 2). The decay ratio is around 50% before 30 min of an ISO 834-1 fire.

Lakhani & Hofmann [19] expanded their study in 2024 by completing a numerical investigation on concrete cone failure during fire exposure using Ansys FE software (version 2022 R2). For groups of anchors, the influence of concrete spacing on capacity was quantified. These results showed that EN 1992-4 [2] is very conservative for low fire durations (<60 min). Furthermore, theoretical predictions (established for h_ef_ = 50 mm) were close to those by other authors using MASA [17,26].

**Figure 3:**
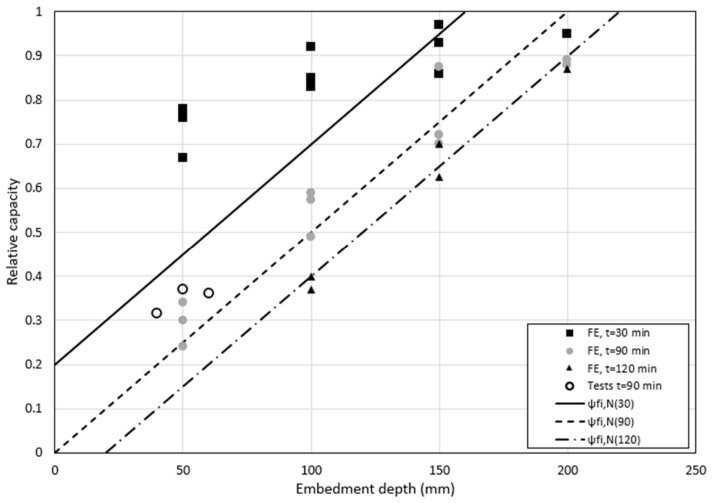
Variation of load capacity versus embedment depth determined by Periskic [26] through tests and simulation.

In 2009, Periskic [26] reinforced the theoretical predictions for tensile concrete cone failure using MASA. The quasi-linear capacity increase with embedment depth (h_ef_) established by Reick in 2001 [17] was confirmed. Using advanced empirical modeling, Periskic proposed three analytical relationships relating to embedment to describe the capacity at 30 min, 90 min and 120 min (Figure 3).

(1) $$\Psi_{fi,N(30)}=0.2+\frac{h_{ef}}{200}\leq 1\;for\;t\leq 30\;min$$

(2) $$\Psi_{fi,N(90)}=\frac{h_{ef}}{200}\leq 1\;for\;30\;min<t\leq 90\;min$$

(3) $$\Psi_{fi,N(120)}=\frac{h_{ef}}{200}-0.1\leq 1\;for\;90\;min<t\leq 120\;min\;\left(with\;h_{ef}\geq 20\;mm\right)$$

where:

- $N_{u,t}$ is the ultimate load at high temperature.
- $k_{1}$ is the concrete cone coefficient (equal to 15.5).
- $f_{c}^{*}$ is the equivalent concrete strength at a given time during fire exposure.
- $h_{ef}$ is the embedment depth.
- $f_{c,i}$ and $A_{i}$ are the concrete strengths and lateral areas of the different slices (referenced by ‘i’).
- n is the number of slices along the cone.
- A is the lateral area of the cone.

**Figure 4:**
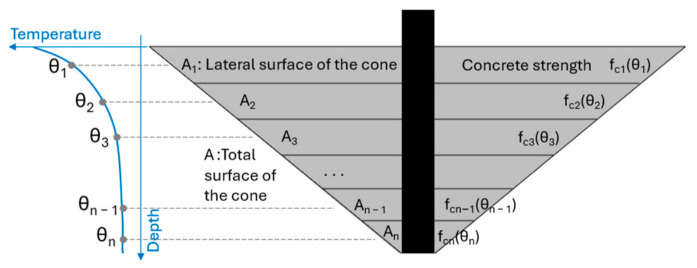
Segmentation of the cone into several slices and thermal profile measured along the anchor line for Hlavička & Lublóy [18].

Other authors have proposed descriptions that depend on temperatures along the anchorage length rather than fire duration. In 2018, Hlavička and Lublóy [18] proposed an analytical method based on temperature (Equation (4)). Prediction of tensile concrete cone capacity relies on an integration of the cone surface by adding the contribution of each slice presenting different temperatures (Figure 4). The concrete mechanical properties used for the integration are: compressive strength $\sqrt{f_{c}}$, tensile strength $f_{t}$, and Young’s modulus with fracture energy $\sqrt{EG}$ in accordance with the theoretical description of the concrete cone failure initially proposed by Bažant & Prat [27]. Theoretical predictions were compared with physical test data obtained using electric heating. The proposed method is biased toward to the portion of the cone nearest to the surface, which is the hottest when the concrete surface is exposed to fire, and does not recognize the fact that failure is controlled by crack propagation from the load-transfer mechanism at the deeper portion of the embedment to the surface as described by Sawade [28].

(4) $$N_{u,t}=k_{1}*\sqrt{f_{c}^{*}}*h_{ef}\;where\;f_{c}^{*}=\sum_{i=1}^{n}f_{c,i}*\frac{A_{i}}{A}$$

where:

- $N_{u,t}$ is the ultimate load at high temperature.
- $k_{1}$ is the concrete cone coefficient (equal to 15.5).
- $f_{c}^{*}$ is the equivalent concrete strength at a given time during the fire.
- $h_{ef}$ is the embedment depth.
- $f_{c,i}$ and $A_{i}$ are the concrete strengths and lateral areas of the different slices (referenced by ‘i’).
- n is the number of slices along the cone.
- A is the lateral area of the cone.

In 2024, Robson [21] proposed a temperature-based design method for tensile concrete cone failure (Equation (5)) after performing advanced modeling and tests on headed anchors in a gas chamber and using radiant heating panels. This work demonstrated that the failure mechanism of the cone is controlled by a crack initiating from the head of the anchor and propagating towards the concrete free surface. In agreement with Bažant & Prat [27] and Sawade [28], this crack propagation phenomenon became unstable at around 40% of the idealized cone’s hypotenuse. Robson found that the cone angle (~35°) between the cone slope and the horizontal direction may be conservatively considered at high temperature. Robson’s comparison of existing research studies revealed considerable scatter in the reported variation of fracture energy with temperature. While some authors consider it increasing, others consider it decreasing as temperature rises [21,29]. Fracture energy has also been used by Bamonte [30] to directly calculate load-bearing capacity under fire conditions using a temperature-dependent formulation proposed by the Model Code [31]. Other authors, e.g., those in Ref. [32], have quantified the tensile energy for different concrete formulations. As in other research studies, Robson observed a substantial decrease in concrete cone capacity in the early stages of an ISO fire. The proposed model is expressed as a reduction coefficient theoretically determined using fracture mechanics principles relying partially on stress intensity factors defined by Irwin [33] and validated through tests and numerical analysis. The concrete mechanical property governing the failure is $\sqrt{EG}$, which can be expressed as proportional to $f_{t}$ [34] or $\sqrt{f_{c}}$ (used as a proxy in code practice).

(5) $$k_{red}=\frac{N_{Fire}}{N_{20{}^{\circ}C}}=\sqrt{\frac{{\left(K_{I}^{2}+K_{II}^{2}\right)}_{20{}^{\circ}C}}{{\left(K_{I}^{2}+K_{II}^{2}\right)}_{Fire}}}*\sqrt{\frac{E\left(T\left(\gamma_{cr}\right)\right)}{E\left(20\;{}^{\circ}C\right)}}*\sqrt{\frac{G_{F}\left(T\left(\gamma_{cr}\right)\right)}{G_{F}\left(20\;{}^{\circ}C\right)}}$$

where:

- $k_{red}$ is the reduction factor under fire.
- $N_{Fire}$ and $N_{20{}^{\circ}C}$ are the load capacities respectively under fire and at ambient temperature.
- $E\left(T\right)$ and $E\left(20\;{}^{\circ}C\right)$ are the Young modulus respectively under fire and at ambient temperature.
- $G\left(T\right)$ and $G\left(20\;{}^{\circ}C\right)$ are the fracture energies respectively under fire and at ambient temperature.
- $K_{I}$ and $K_{II}$ are the stress intensity factors associated with crack propagation modes I and II.
- $\gamma_{cr}$ is the position along the diagonal at which the propagation becomes unstable.

The current limitations in the existing scientific background are:

(1) The lack of formalized design methods based on temperature (instead of a prescriptive formula based on time and embedment depth h_ef_). Only Robson and Hlavička proposed temperature-based approaches.
(2) All studies exclusively studied one-sided fire exposure on the top concrete surface. The influence of heating orientations is not described.
(3) Parameters such as concrete strength, cracked concrete, group effects, and edge effect are not always covered. Lakhani and Periskic covered group and edge effects. Robson analyzed the effect of anchor technology.

### 2.2. Existing Design Method

The informative Annex D of EN 1992-4 [2] provides a prescriptive equation providing capacities for a fire before 90 min ($N_{Rk,c,fi(90)}^{0}$) and after 90 min ($N_{Rk,c,fi(120)}^{0}$) [16] as given in Equations (6) and (7). This equation is simple to use for design; however, the method is not applicable for fire durations longer than 120 min and is overconservative for short fire durations (30 min, 60 min) when compared to the research results summarized in Section 2.1. Furthermore, based on the available background research [17], the equation seems to have been developed based only on one-sided fire exposure (that is, with the fire acting on the surface from which the anchor is installed).

(6) $$N_{Rk,c,fi(90)}^{0}=\frac{h_{ef}}{200}\cdot N_{Rk,c}^{0}\;for\;fire\;exposure\;up\;to\;90\;min$$

(7) $$N_{Rk,c,fi(120)}^{0}=0.8\cdot \frac{h_{ef}}{200}\cdot N_{Rk,c}^{0}\;for\;fire\;exposure\;between\;90\;\min\;and\;120\;min$$

where:

- $h_{ef}$ is the effective embedment depth.
- $N_{Rk,c}^{0}$ is the characteristic resistance of a single fastener in cracked concrete C20/25 under ambient temperature.

This prescriptive equation depends solely on the embedment depth. For large embedment depths (at or above h_ef_ = 200 mm), no capacity reduction is predicted for up to 90 min of fire exposure. According to the theoretical temperature profiles provided by the EN 1992-1-2 [9] and thermal properties for concrete with an ISO 834-1 fire [16], the temperature at a depth of h_ef_ = 200 mm remains close to 20 °C after 90 min. Implicitly, this indicates that the mechanical degradation of the concrete at high temperatures near the exposed surface does not significantly affect the capacity of the system. Instead, the deeper (and cooler) areas appear to govern the failure mechanism, which is consistent with the physical interpretations based on fracture mechanics that have been conducted by Sawade, Reick, and Robson.

### 2.3. Outputs from Literature Review

Section 2.1 reveals a research gap in thermal configurations other than single-sided heating and key variables (including group effects, edge distance, and different anchoring technologies) to characterize tensile concrete cone capacity under fire conditions. A clear impact that has been overlooked in several studies is how thermal gradients interact with crack propagation and load transfer mechanisms. This item is essential to propose a temperature-based method that may be applied for different fire scenarios.

**Figure 5:**
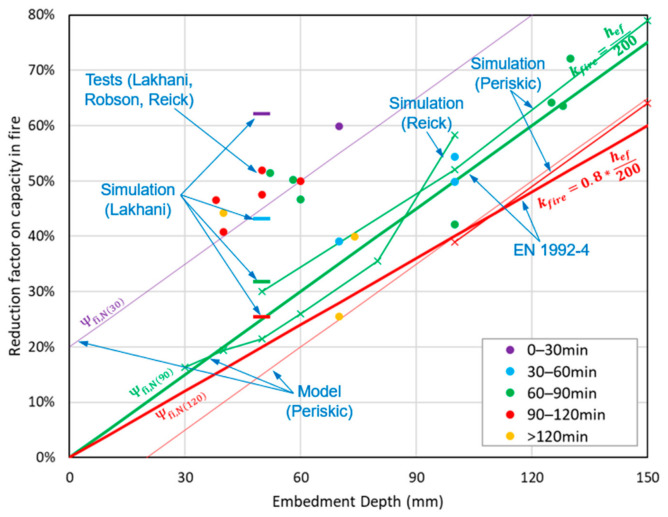
Compiled TEST data (Reick, Lakhani, Robson) and SIMULATION (Reick, Lakhani, Robson, Periskic) on the same plot with an EN 1992-4 design.

Figure 5 presents compiled test data (Reick, Lakhani, Robson) and numerical studies (Reick, Lakhani, Robson, Periskic) plotted alongside EN 1992-4 design predictions. The test data are shown as discrete points, while numerical simulations and analytical models are represented by solid lines. The compiled test data are highly scattered, with data points for different fire ratings superimposed within the same cloud and above numerical predictions. Nevertheless, the simulations enable the identification of clear linear trends of increasing resistance with increasing embedment depth.

A trend emerges from the two statements below, which are shared by several authors:

(i) There is a rapid reduction in the capacity of shallower embedment depths at the early stages of the fire exposure as the temperature increases, typically within the first 30 min.
(ii) For large embedment depths (close to 200 mm), the cone resistance remains close to 20 °C capacity when EN 1992-4 is applied for up to 90 min of exposure. Consistently, MASA simulations also converge towards a 20 °C capacity for these embedment depths.

**Figure 6:**
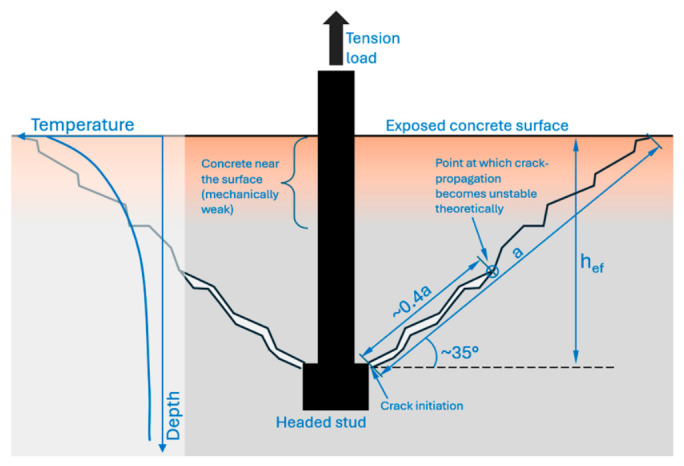
Sketch of crack formation during concrete cone failure under fire exposure from the top.

Statement (i) implies that, although the temperature at the exposed surface is significantly elevated, the temperature at the embedment depth remains near 20 °C. Thus, failure of shallower embedments seems to be highly influenced by the layer of concrete weakened by high temperatures. By contrast, the second statement (ii) indicates that the hot layer near the exposed surface (mechanically weak) does not seem to affect the overall capacity for larger embedments. Instead, resistance is maintained by the deeper area where temperatures for embedment depths around 200 mm remain close to 20 °C even after 90 min. of fire exposure (Figure 6).

## 3. Methodology

### 3.1. Test Program

To determine a temperature-based design method for tensile concrete cone failure of anchors subjected to fire conditions, a parametric experimental analysis was conducted. The test program (Table 1) was defined by sequentially modifying the influence of cracked concrete, heating orientation, anchor group effects, concrete strength and anchor technology to quantify their effects during fire exposure.

Anchors were post-installed within the crack, and the crack width was maintained at a constant 0.3 mm width during heating before performing residual tensile tests using the same procedure as presented in [11]. The crack was generated using expansion wedges positioned vertically left and right from the anchor in a portion of the slab which contained lateral thin steel sheets on the side to force the crack path through the width of the cross-section. The opening of the crack was measured with a horizontal displacement sensor near the anchor. A crack thickness of 0.3 mm was achieved by inserting the wedges with a hammer. Both wedges were positioned at a distance greater than 150 mm from the radiant panel and were protected from heat exposure.

Group tests were performed on two equally loaded headed anchors spaced at 100 mm. The bottom-heating tests were performed on concrete slabs with a thickness of 200 mm. The concrete cover between the bottom point of the anchor (embedded at 92 mm) and the exposed concrete surface of the concrete was thus 108 mm.

### 3.2. Anchor Description

Anchor parameters were chosen to produce concrete cone failure as the controlling failure mode during reference conditions in room-temperature concrete and during fire tests. Most of the tests were performed on carbon-steel cast-in headed anchors with a diameter of 16 mm and an embedment depth of 100 mm. Tests performed on C50/60 concrete, cracked concrete, and from heating of the bottom surface of the slab using an ISO 834-1 fire were instead performed using HDA-P undercut anchors with a diameter of 16 mm. To achieve concrete cone failure in C50/60, the embedment depth was limited to 57 mm. For bottom heating tests, the embedment depth was 92 mm, allowing temperature to increase at the lower part of the anchor while still targeting cone failure. Cracked concrete tests were performed on 20/25 concrete with an embedment depth of 77 mm.

To investigate the influence of the load transfer mechanism, HUS4 concrete screws with a diameter of 16 mm and an embedment depth of 100 mm were also tested. Unlike headed anchors and undercut HDA-P anchors, for which the load transfer from the steel to the concrete is localized at the bottom part of the anchor, concrete screws exhibit a continuous load transfer along the embedment depth via the screw threads.

### 3.3. Concrete Characterization

The tests in Table 1 lines 1 to 10 were performed on concrete slabs with dimensions of 2400 mm × 600 mm × 300 mm. The tests from lines 11 to 15 were performed on slabs with dimensions of 1400 mm × 1200 mm × 200 mm. Each slab was cured for a duration longer than 3 months to mitigate the possibility of concrete spalling during fire exposure. Concrete compressive strengths were characterized before testing in accordance with EN 12390-3 [35]. The C20/25 concrete used for lines 1 to 10 had a compressive strength (for 150 mm × 150 mm × 150 mm cubes) of f_c_ = 34.4 MPa. The C20/25 concrete used for lines 11, 14 and 15 had a compressive strength (for 150 mm × 150 mm× 150 mm cubes) of f_c_ = 33.0 MPa. The C50/60 concrete used for lines 12 and 13 had a compressive strength (on 150 mm × 150 mm × 150 mm cubes) of f_c_ = 63.0 MPa.

### 3.4. Test Procedure with Radiant Panels

**Figure 7:**
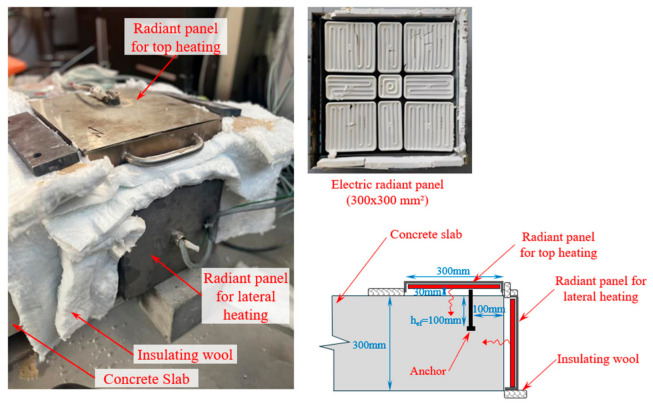
Radiant heating panel apparatus used on concrete specimens.

All tests except those in line 11 were performed using radiant panel heating devices powered at 380 V. The radiant panels presented a surface of 300 mm × 300 mm composed of several ceramic resistances encased in a stainless-steel frame (Figure 7). The resistances were positioned repeatably 35 mm from the concrete surface and heat was mostly applied though radiation. The surrounding areas around the panel were insulated using Isofrax wool. The panel was regulated to follow a linear slope up to a target setpoint which yielded a temperature on the concrete surface generally in the range of 400–600 °C depending on the tests. The heated boundary condition was maintained for a duration between 3.5 and 8 h to generate a thermal gradient through the thickness of the concrete. This gradient generally presented a linear trend since the conditions were close to quasi-static thermal transfer, as opposed to a transient regime for gas heating.

### 3.5. Test Procedure with Gas Heating ISO 834-1 (Line 11)

**Figure 8:**
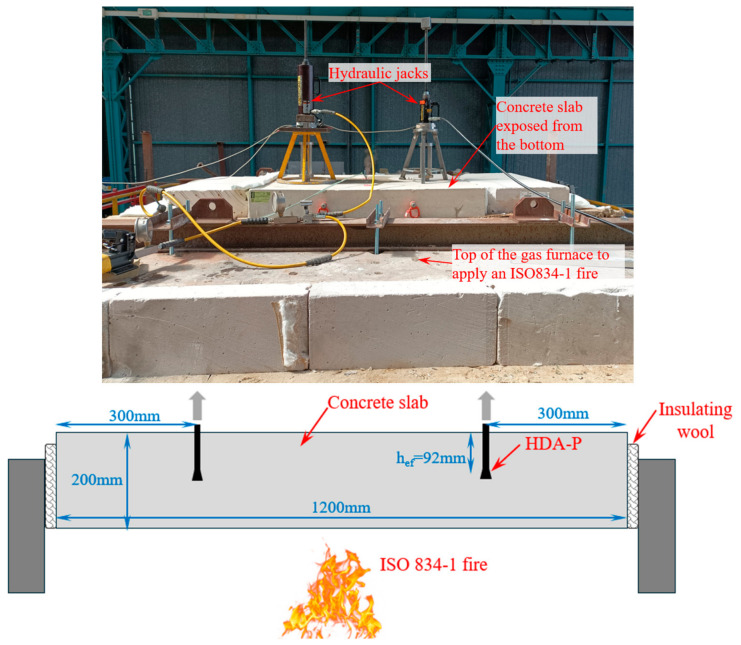
Tension applied to anchors during heating from the bottom.

Bottom-heating tests were performed with an ISO 834-1 fire to ensure that the thermal diffusion would be sufficient to induce a reduction in anchor capacity. The C20/25 concrete slab (1400 mm × 1200 mm × 200 mm) was positioned on top of a 4 m × 3 m horizontal furnace complying with the ISO 834-1 conventional time–temperature curve (Figure 8). For each test, two anchors were tested using manual hydraulic jacks positioned on top of the slab. Just like the electrically heated tests, the load was applied after a targeted fire duration.

### 3.6. Instrumentation

**Figure 9:**
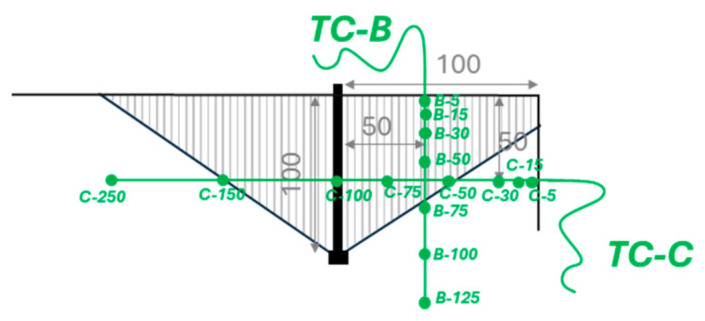
Position of the thermocouples for tests performed with radiant panels.

For each test conducted using radiant panel heating, thermocouple lines were installed to measure the temperature profile through the slab thickness (Figure 9). In the vertical direction, temperatures were measured at depths of 5, 15, 30, 50, 75, 100, and 125 mm (referenced ‘TC-B’) along a line positioned 50 mm away from the anchor. For the tests involving lateral heating, horizontal temperature measurements were performed at 5, 30, 50, 75, 100, 150, and 250 mm from the heated surface (referenced ‘TC-C’) along a line positioned 50 mm below the top concrete surface.

## 4. Design Method

### 4.1. Proposed Design Method

The design method proposed in TR069 [36] to determine anchor capacity under fire exposure consists of applying a temperature-dependent reduction factor k_c_(θ) to the reference average capacity ($N_{Rm,c}^{0}$) at 20 °C (e.g., ambient temperature). This reduction factor depends solely on the temperature at the deepest point of the embedment depth, which is assumed to be representative of the crack initiation zone governing the formation of the concrete cone. The average load capacity under fire at a given time t of exposure is expressed as:

(8) $${N_{Rm,c,\;FIRE}^{0}(t)=k_{c}\left(\theta (t)\right)\cdot N}_{Rm,c}^{0}$$

(9) $$k_{c}\left(\theta \right)=\frac{1.1}{1+\frac{\theta -20}{135}}-0.1$$

where:

- $N_{Rm,c}^{0}$ is the average concrete cone capacity calculated with Equation (10).
- $N_{Rm,c,FIRE}^{0}$ is the average load capacity under fire at a given time.
- θ is the temperature at the deepest part of the embedment depth at a given time.
- $k_{c}$ is the reduction factor for concrete cone failure.

**Figure 10:**
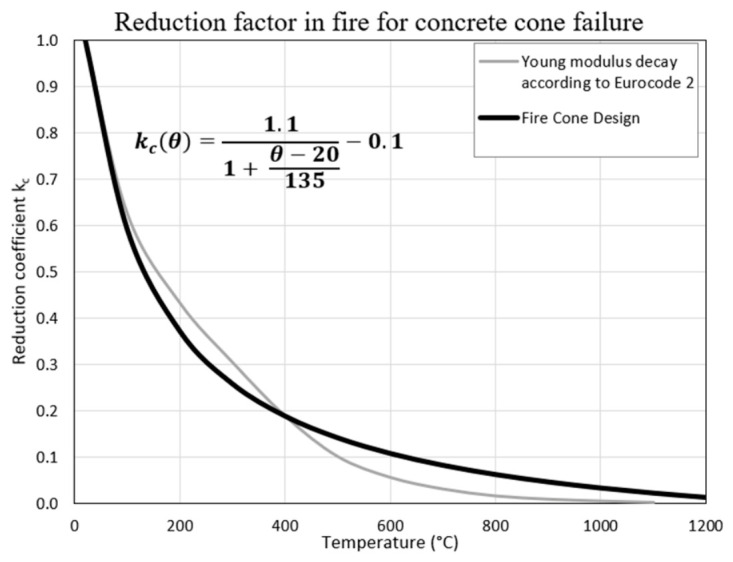
Reduction ratio, $k_{c}$, for effects of temperature at the effective embedment depth on concrete cone capacity.

The function defined in Equation (9) was derived empirically and exhibits a rapid decay in the early stages of fire exposure (Figure 10). This behavior is consistent with experimental and numerical observations from [17,21,25,26]. Furthermore, the trend closely represents the decay of the Young’s modulus implicitly described in 1992-1-2 [9], in terms of the variations of compressive strength and strains with temperatures (Figure 10).

### 4.2. Comparison with Eurocode

**Figure 11:**
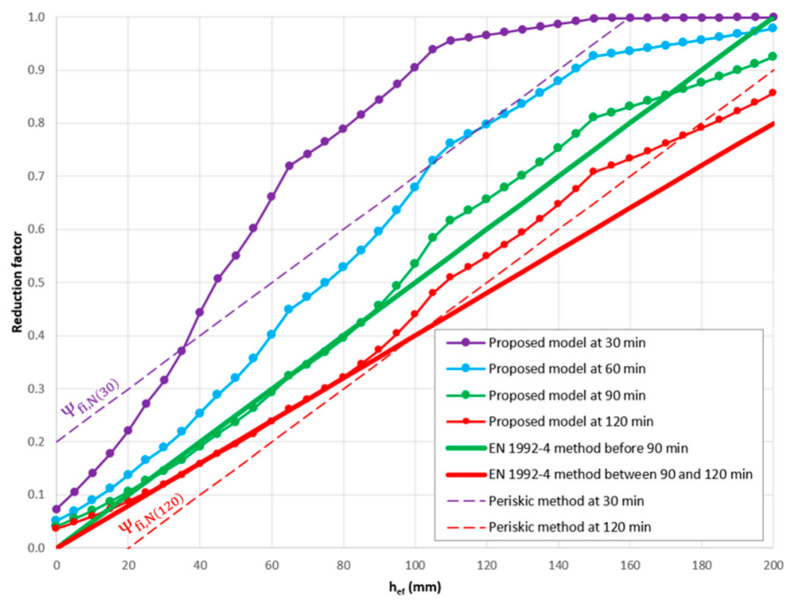
Comparison of the proposed design method with the test data (Reick, Lakhani, Robson), previous methods (Reick, Periskic), and EN 1992-4.

Figure 11 compares the proposed design method with available experimental results (Reick, Lakhani, Robson), previously published methods (Reick, Periskic), and the design predictions of EN 1992-4. The temperature profiles required to predict capacities were determined using the thermal material properties available from EN 1992-1-2 (variations of thermal conductivity, specific heat, and density with temperature). The analyses were conducted for a configuration with fire exposure from the top concrete surface (in which the anchor is embedded) following the conventional ISO 834-1 fire curve.

At 120 min of fire exposure, the proposed method yields results similar to, or slightly higher than, those predicted by EN 1992-4. The method also follows a similar trend identified at the end of the background analysis, with the capacity being close to zero for small embedment depths and converging towards the 20 °C capacity for large embedment depths. Unlike EN 1992-4, the proposed method allows for the determination of anchor capacities for intermediate fire durations, such as 30 min and 60 min, since it is explicitly based on temperatures rather than exposure time. The minor oscillations noticeable on the prediction curves are caused by temperature profiles calculated according to EN 1992-1-2 linked to the vaporization plateau of water near 100 °C. The transformation from temperature to capacity using the function in Equation (9) captures part of these variations. The latent heat absorption is indirectly produced by a peak of equivalent specific heat as described in EN 1992-1-2. The calculated effect of water vaporization generally underestimates the temperature plateau measured in tests. The theoretical temperatures are thus overestimated, yielding conservative design. In addition, water vaporization caused by the discontinuity of the concrete by the anchor borehole substantially retards the increase in temperature locally around the anchor. This is described in Section 5.8.

## 5. Results

### 5.1. Overview of Parametric Tests

**Figure 12:**
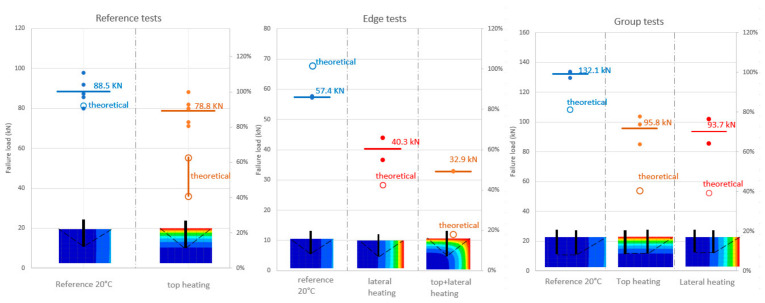

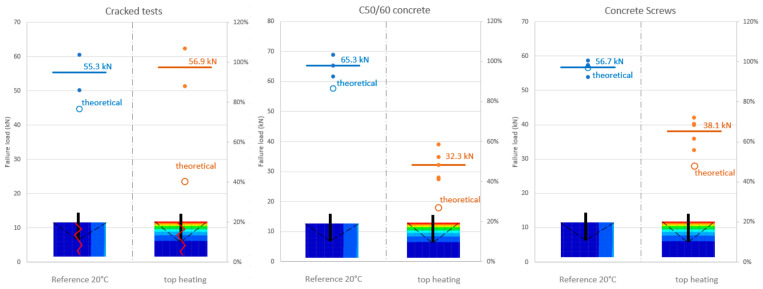
Summary of the parametric tests: predicted and measured capacities at ambient temperature and at high temperature.

The parametric investigation was performed using radiant panels to assess the influence of the different parameters. Table 2 and Figure 12 present a summary of the measured and predicted capacities at ambient temperature and at high temperatures. For each parameter, the analysis in this section includes: the predicted and measured capacities at ambient temperature (from EN 1992-4 design predictions and test results), the measured and the predicted capacities at high temperatures (from the measured temperatures), and qualitative observations on the concrete cone geometries after failure. The theoretical capacity in the last column was determined by applying the design method (Equations (8) and (9)) using the measured temperature at the embedment depth.

### 5.2. References Tests

For tests performed at ambient temperature (line 1 of the test program), the predicted concrete cone capacity was calculated using Equation (10) [37,38]. It should be noted that EN 1992-4 determines the concrete cone capacity using a compressive strength obtained from cylinders rather than cubes, thus yielding capacities multiplied by a factor of $\sqrt{0.8}=0.9$.

(10) $$N_{Rm,c}^{0}=k_{1}\cdot \sqrt{f_{c}}\cdot h_{ef}^{1.5}=15.5*\sqrt{0.95*34.4}*100^{1.5}=88.6\;kN$$

where:

- $N_{Rm,c}^{0}$ is the average load capacity.
- $k_{1}$ is the coefficient equal to 15.5 to account for an average (not characteristic) capacity for headed bolts in uncracked concrete.

$f_{c}$ is the compressive strength of concrete for a 200 mm cube (the 0.95 coefficient converts compressive strength from a 150 mm cube to a 200 mm cube).

- $h_{ef}$ is the embedment depth.

The average capacity obtained from five repeated reference tests at 20 °C was 88.5 kN (with a coefficient of variation of 7.6%) with good agreement between the prediction and experimental results. The tests were conducted under static loading using a displacement-controlled hydraulic jack until failure.

**Figure 13:**
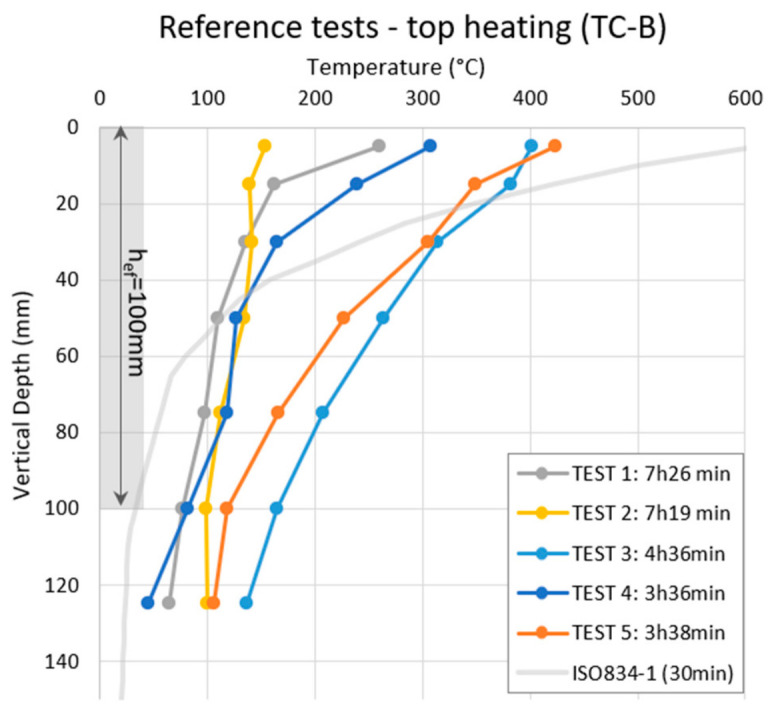
Temperature profiles during loading for the tests heated from the top using radiant panels and comparison with the theoretical thermal profile at 30 min of ISO 834-1 heating.

Figure 13 presents the temperature profiles measured inside the concrete during loading along the thermocouple line TC-B (Figure 9) for heating durations ranging between 3 h 38 min and 7 h 26 min. The theoretical thermal profile at 30 min for an ISO 834-1 fire is also plotted in Figure 13 for a comparison of the thermal gradients. These temperatures were calculated using the method presented in Section 5.8. Due to differences in heating rates and target temperatures, the resulting thermal profiles exhibit noticeable scatter. The temperature at the embedment depth of the anchor varied between 82 and 160 °C. Applying the proposed temperature-based model described above yields predicted capacities between 36.1 and 55.3 kN.

The average measured capacity under heated conditions (line 2 of the test program) was 78.8 kN, with a coefficient of variation of 8.7%, corresponding to a reduction close to 11% compared to that under ambient temperature. This reduction is relatively mild compared with those in other studies. For example, Robson [21] reported decays closer to 50% for similar test conditions. The theoretical maximal prediction (55.3 kN) was conservative relative to the experimentally observed average at a high temperature (78.8 kN).

Observation of cone geometries post-test did not reveal notable change in geometry compared to that under ambient temperature. These results support the assumption that the cone angle does not change significantly with temperature within the investigated range of parameters.

### 5.3. Effect of Edge Distance

At ambient temperature, the reduction in capacity due to edge effects was calculated in accordance with EN 1992-4 using the ratio of areas of the concrete surface with an edge limitation ($A_{c,N}$) and with a fully developed cone ($A_{c,N}^{0}$).

(11) $$\frac{A_{c,N}}{A_{c,N}^{0}}=\frac{3h_{ef}\cdot (1.5h_{ef}+c_{1})}{9h_{ef}}=\frac{300*(150+100)}{300*300}=0.83$$

(12) $$N_{Rm,c}=\frac{A_{c,N}}{A_{c,N}^{0}}\cdot N_{Rm,c}^{0}=0.83*88.6=73.8\;kN$$

where:

- $A_{c,N}$ is the concrete surface including the edge effect.
- $A_{c,N}^{0}$ is the concrete surface for a fully developed cone.
- $h_{ef}$ is the embedment depth.
- $c_{1}$ is the edge distance.

The multiplication factor induced by the edge distance (0.83) produced a capacity of 73.8 kN at ambient temperature, which was higher than the value of 57.4 kN obtained experimentally.

**Figure 14:**
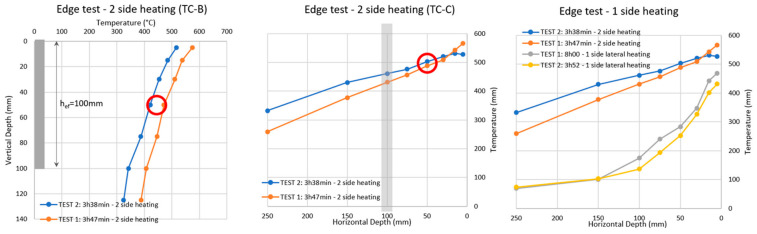
Temperature profiles in the vertical and horizontal directions for a test with a small edge distance.

High-temperature tests were performed with lateral heating from one side [lateral edge] and from two sides [lateral edge and top of the concrete] in order to induce higher temperatures along the anchor embedment length (Figure 14). For one-sided lateral heating, the top of the slab was thermally isolated, resulting in isotherms parallel to the anchor axis. The temperature at the embedment of the headed anchor reached approximately 155 °C, corresponding to a multiplication factor of 0.45 and a predicted capacity of 33.2 kN. This prediction remains conservative when compared with the average measured capacity (40.3 kN). For two-sided heating, the average temperature at the embedment depth of h_ef_ = 100 mm increased to approximately 375 °C. The associated multiplication factor of 0.20, applied to the theoretical capacity at 20 °C (73.8 kN), resulted in a predicted high-temperature capacity of 14.8 kN.

### 5.4. Effect of Group of Anchors

**Figure 15:**
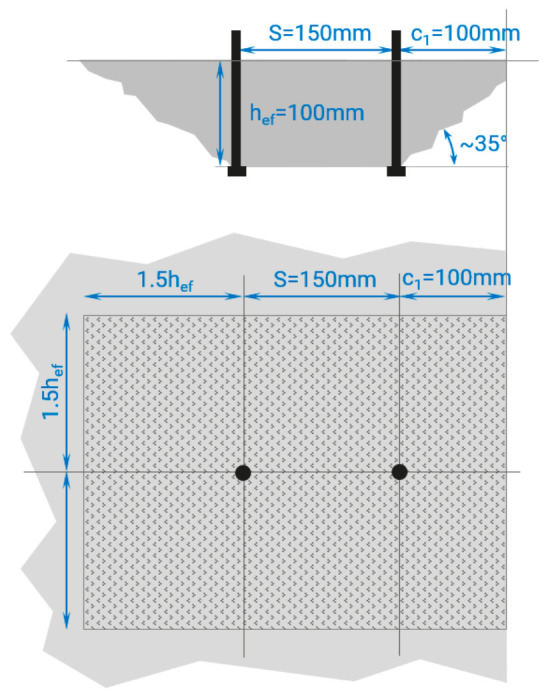
Plan-view dimensions of anchor group tests.

The value at ambient temperature of the capacity of a group of two anchors (Figure 15) was calculated in accordance with EN 1992-4 by multiplying the capacity by the ratio of areas of the concrete surface associated with the group ($A_{c,N}$) and with a fully developed cone ($A_{c,N}^{0}$).

(13) $$\frac{A_{c,N}}{A_{c,N}^{0}}=\frac{3h_{ef}\cdot (1.5h_{ef}+s+c_{1})}{9h_{ef}}=\frac{300*(150+150+100)}{300*300}=1.33$$

(14) $$N_{Rm,c}=\frac{A_{c,N}}{A_{c,N}^{0}}\cdot N_{Rm,c}^{0}=1.33*88.6=118.1\;kN$$

where:

- $A_{c,N}$ is the concrete surface of the group of anchors.
- $A_{c,N}^{0}$ is the concrete surface for a fully developed cone.
- $h_{ef}$ is the embedment depth.
- $c_{1}$ is the edge distance.
- s is the distance between both anchors.

The theoretical group multiplication factor of 1.33 leads to a predicted ambient temperature capacity of 118.1 kN, which is slightly lower than the experimentally measured average capacity of 132.1 kN, indicating that the formula used for ambient temperature design is conservative when applying the group influence factor from EN 1992-4 for these tests.

**Figure 16:**
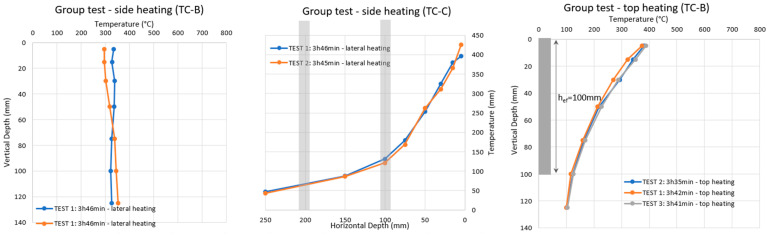
Temperature profiles in the vertical and horizontal directions for group tests.

High-temperature tests were performed with lateral heating and with top heating (Figure 16). For one-sided lateral hearing, the top surface of the concrete slab was insulated, resulting in isotherms approximately parallel to the headed anchor axis. The temperature at the embedment depth of the most exposed anchor was 127 °C, while the temperature at the embedment depth of the second anchor, located further away from the exposed surface, was approximately 60 °C. Considering only the most critical (hottest) anchor, a multiplication factor of 0.51 was obtained, yielding a predicted capacity of 60.7 kN. This theoretical capacity was conservative compared to the average measured capacity of 93.7 kN. For the tests performed with top heating, the temperature at the bottom of the headed bolt was approximately 121 °C, corresponding to a fire multiplication factor of 0.53 and a calculated load capacity of 62.5 kN. This value is again conservative in comparison to the experimentally measured average capacity (95.8 kN). Overall, the proposed design method consistently underestimates the experimentally measured capacities for the tested anchor groups under fire exposure.

### 5.5. Effect of Cracked Concrete

The theoretical value at ambient temperature of the capacity of anchors installed in cracked concrete was determined using Equation (15).

(15) $$N_{Rm,c,crack}=\psi_{c}\cdot k_{1}\cdot \sqrt{f_{c}}\cdot h_{ef}^{1.5}=0.7*15.5*\sqrt{0.95*34.4}*77^{1.5}=41.9\;kN$$

where:

- $N_{Rm,c,crack}$ is the average load capacity in cracked concrete.
- $\psi_{c}$ is the factor accounting for crack effects equal to 0.7.
- $k_{1}$ is the coefficient equal to 15.5 to account for an average (not characteristic) capacity for headed bolts in uncracked concrete.
- $f_{c}$ is the compressive strength of concrete for a 200 mm cube (the 0.95 coefficient converts compressive strength from a 150 mm cube to a 200 mm cube).
- $h_{ef}$ is the embedment depth.

**Figure 17:**
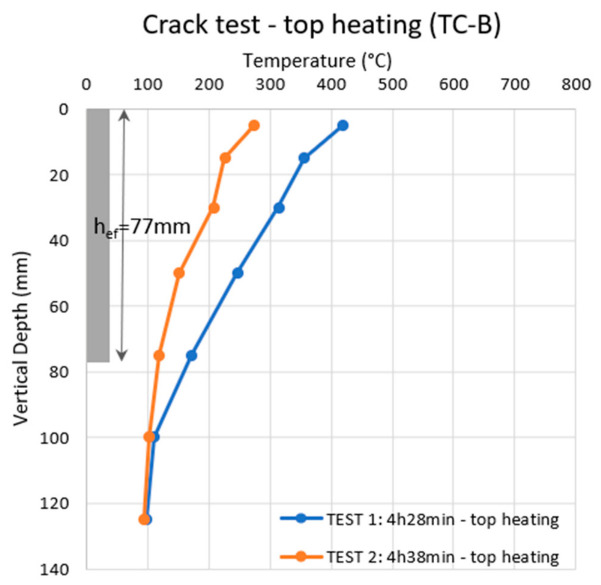
Temperature profiles for tests in cracked concrete.

High-temperature tests on cracked concrete were performed with one-sided heating from the top. The measured temperature profiles produced large scatter (Figure 17). As reported in the literature, cracks may contribute to mass water transport within concrete, which can substantially affect temperature distribution (especially around 100 °C due to water vaporization). The temperature at the bottom of the headed anchor was approximately 110 °C, corresponding to a decay of 54% and a predicted capacity at high temperature equal to 22.6 kN. This value is very conservative compared with the experimentally measured average capacity of 56.9 kN at a high temperature. There was no substantial reduction from the capacities at ambient temperature to the capacities at a high temperature in cracked concrete. One likely explanation is that the temperature fields were measured inside uncracked concrete rather than along the anchor or within the crack. Previous experience has shown that cracks tend to create a more uniform temperature distribution along an anchor, potentially cooling the locations where the temperature exposure is highest. For this reason, the temperature may have been sufficiently low at the bottom of the anchor to preclude concrete cone strength degradation. Based on these results, no additional reduction in capacity beyond the cracked concrete factor $\psi_{c}=0.7$ appears necessary for fire design. However, even when cracked concrete is present as a design condition (either in ambient or fire conditions), because the cracks may not necessarily pass through anchors, it is recommended to use the lesser of the ambient cracked concrete capacity and the uncracked concrete reduction under fire conditions.

**Figure 18:**
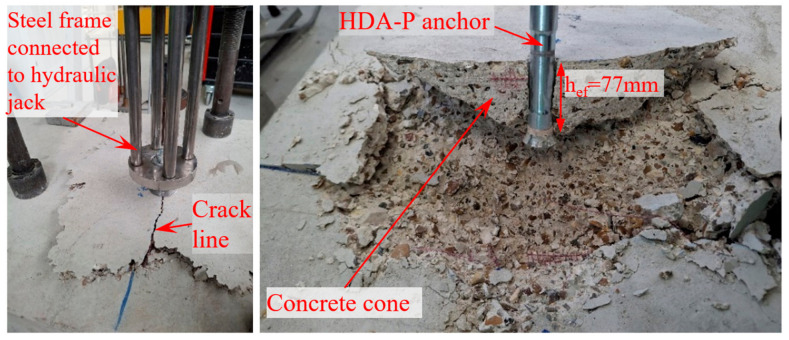
Photographs of failure.

Post-test observations of the geometry of the concrete cones confirm that the crack indeed passed through the anchor line (Figure 18), and no variations in the cone geometry were identified under the effect of temperature.

### 5.6. Effect of Concrete Strength

Tests on high-strength concrete were conducted using HDA-P M16 anchors with an embedment depth of 57 mm in order to avoid steel failure. The ambient-temperature concrete cone capacity was calculated as:

(16) $$N_{Rm,c}^{0}=15.5\sqrt{f_{c}}\cdot h_{ef}^{1.5}=15.5*\sqrt{0.95*63}*57^{1.5}=51.6\;kN$$

where:

- $N_{Rm,c}$ is the average load capacity.
- $\psi_{c}$ is the factor accounting for crack effects equal to 0.7.
- $k_{1}$ is the coefficient equal to 15.5 to account for an average (not characteristic) capacity for headed bolts in uncracked concrete.
- $f_{c}$ is the compressive strength of concrete for a 200 mm cube (the 0.95 coefficient converts compressive strength from a 150 mm cube to a 200 mm cube).
- $h_{ef}$ is the embedment depth.

At ambient temperature, this calculated capacity (51.6 kN) was lower than the experimentally measured average capacity (65.3 kN), again indicating conservatism of the proposed design approach. Tests at a high temperature were performed with top-heating. At a depth of h_ef_ = 57 mm, the temperature reached approximately 220 °C, corresponding to a multiplication factor of 0.34 and a predicted capacity of 17.7 kN. This prediction is conservative compared with the average measured capacity of 32.3 kN.

The multiplication factor between the ambient- and high-temperature capacities was close to 0.50 for C50/60 concrete, and close to 0.90 for C20/25 concrete. This difference may be attributed to the fact that the embedment depth for the C20/25 tests was h_ef_ = 100 mm (instead of 57 mm for C50/60), resulting in a lower temperature at the bottom of the anchors and a higher predicted capacity.

The observation of failure surfaces in higher-strength concrete tests suggests that concrete cones formed at high temperatures exhibited a wider fracture surface than those formed at ambient temperature. Cone formation during testing was accompanied by a sudden popping noise, similar to that observed during concrete spalling. This phenomenon was also reported by Robson [21] and is commonly associated with pore pressure build-up due to water vaporization within the concrete microstructure [39,40,41].

### 5.7. Effect of Fastener Technology

The ambient-temperature capacity for HUS4 concrete screws was calculated using Equation (17).

(17) $$N_{Rm,c}^{0}=\frac{15.5}{12.7}*11\sqrt{f_{c}}\cdot h_{ef}^{1.5}=\frac{15.5}{12.7}*11*\sqrt{0.95*33}*{\left(0.85*95\right)}^{1.5}=54.5\;kN$$

where:

- $N_{Rm,c}^{0}$ is the average load capacity.
- $k_{1}$ is the coefficient equal to 11 to account for a characteristic capacity for concrete screws in uncracked concrete. The ratio 15.5/12.7 allows to pass from a characteristic value to an average value (using the references from headed bolts.
- $f_{c}$ is the compressive strength of concrete for a 200 mm cube (the 0.95 coefficient converts compressive strength from a 150 mm cube to a 200 mm cube).
- $h_{ef}$ is the embedment depth (equal to 0.85 times the nominal length of 95 mm).

The theoretical capacity of 54.5 kN is in accordance with the experimentally measured average capacity (56.7 kN). The temperature at a depth of h_ef_ = 100 mm was approximately 121 °C, corresponding to a multiplication factor of 0.53 and a predicted high-temperature capacity of 28.9 kN. This value remains conservative relative to the measured average capacity of 38.1 kN.

**Figure 19:**
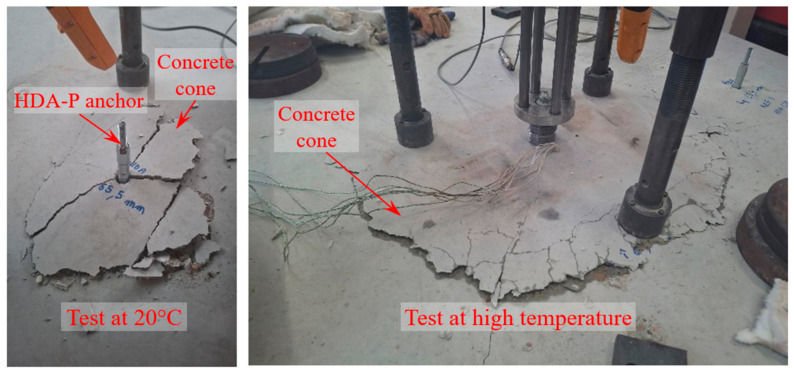
Comparison of the cone geometries for the tests at 20 °C and exposed to a high temperature.

It can be noted that the relative reduction in capacity for concrete screws in C20/25 concrete (from 56.7 to 38.1 kN, corresponding to a ratio of 0.67) is higher than that for headed anchors (from 88.5 to 78.9 kN, corresponding to a ratio of 0.89). This suggests a strong influence of anchor technology, likely related to the fact that the load is more distributed along the anchor embedded length, which may shift the governing failure point higher up and closer to the heated region. While it is difficult to isolate concrete cone failure at high temperatures in organic adhesives, it is believed that this observation likely also applies to organic chemical anchors (Figure 19).

### 5.8. Effect of Bottom Heating

For tests conducted in the gas furnace with bottom heating, the ambient-temperature capacity is calculated by Equation (18).

(18) $$N_{Rm,c}^{0}=15.5\sqrt{f_{c}}\cdot h_{ef}^{1.5}=15.5*\sqrt{0.95*33}*92^{1.5}=76.6\;kN$$

where:

- $N_{Rm,c}^{0}$ is the average load capacity in cracked concrete.
- $k_{1}$ is the coefficient equal to 15.5 to account for an average (not characteristic) capacity for headed bolts in uncracked concrete.
- $f_{c}$ is the compressive strength of concrete for a 200 mm cube (the 0.95 coefficient converts compressive strength from a 150 mm cube to a 200 mm cube).
- $h_{ef}$ is the embedment depth (equal to 100 − 8 = 92 mm for an HDA anchor installed with a drilling depth of 100 mm).

**Figure 20:**
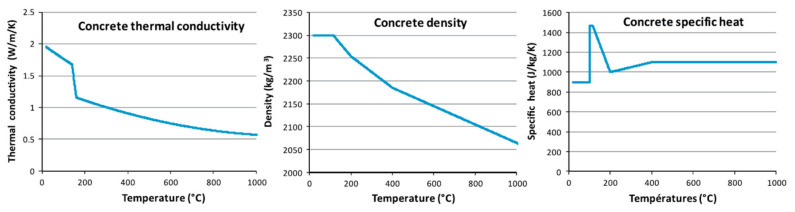
Variations of thermal conductivity, density and specific heat of the concrete with temperatures in accordance with EN 1992-1-2.

The calculated temperatures are determined with the method described in EN 1992-1-2. Conduction is described with a 1D thermal transfer using the Fourier equation. The variation in thermal properties (thermal conductivity, mass density and specific heat) with temperature is presented in Figure 20. The entry flux densities on the concrete exposed and non-exposed surfaces are described by convective (Equation (19)) and radiative (Equation (20)) components.

(19) $$Q_{conv}=h\cdot \left(\theta_{exterior}-\theta_{surface}\right)$$

(20) $$Q_{rad}=\varepsilon \cdot \sigma \cdot \left(\theta_{exterior}^{4}-\theta_{surface}^{4}\right)$$

where:

- $Q_{conv}$ is the convective flux density.
- $Q_{rad}$ is the radiative flux density.
- $\theta_{exterior}$ is the exterior temperature (corresponding to the ISO 834-1 fire for the exposed surface or 20 °C for the non-exposed surface).
- $\theta_{surface}$ is the temperature of the concrete surface.
- h is the convective exchange coefficient (equal to 25 W/m^2^/K for the exposed surface and to 4 W/m^2^/K for the non-exposed surface).
- ε is the emissivity of concrete (equal to 0.7).
- σ is the Stefan–Boltzmann constant equal to 5.67 × 10^−8^ W/m^2^/K^4^.

Table 3 presents the experimentally measured capacities obtained during the ISO 834-1 bottom-heating tests, together with theoretical predictions based on measured and calculated temperature profiles. The multiplication factors shown in Figure 19 are presented using the measured failure load at ambient temperature (55 kN) as a reference. The overall reduction in capacity at different fire durations is evaluated using the proposed temperature-based design method (Equation (8)), rather than the prescriptive EN 1992-4 fire design approach. The required temperatures were calculated using EN 1992-1-2 thermal material properties (specific heat, conductivity, and density).

**Figure 21:**
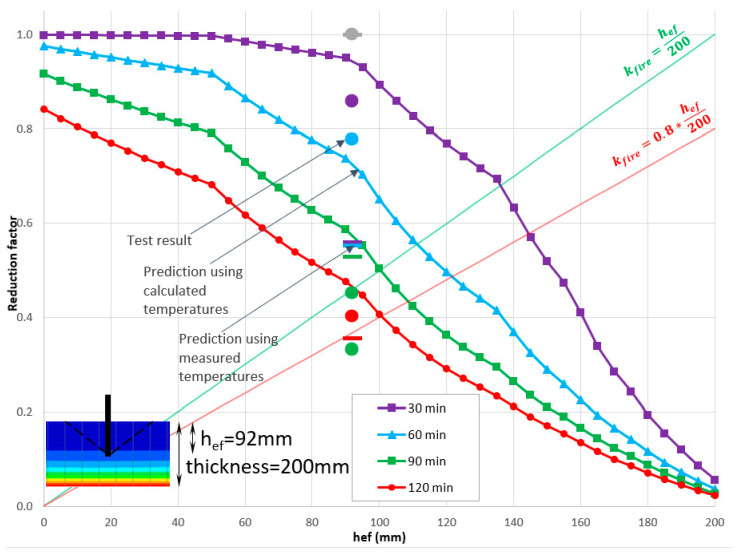
Decay measured through testing and theoretically calculated for the bottom-heating gas tests.

**Figure 22:**
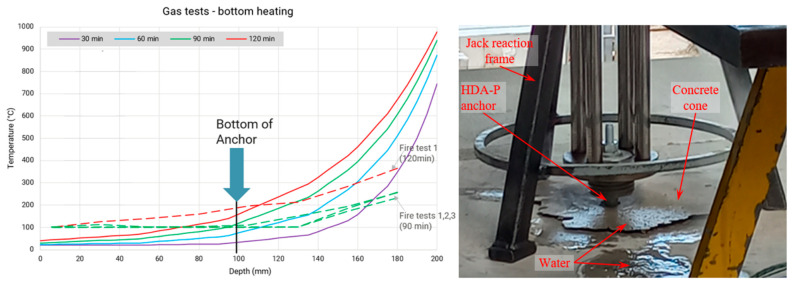
Measured and theoretical temperature profiles for gas heating and a photograph at 30 min during the cone failure on top of the furnace.

All observed failures were governed by concrete cone breakout. The measured capacities generally decrease with fire duration, with the exception of two data points at 90 min, which were higher than those at 120 min (Figure 21). For this reason, the tests at 90 min were repeated in order to assess scatter. Temperatures were measured inside the concrete through the slab thickness, and a substantial water vaporization plateau at approximately 100 °C was measured for each test between 20 and 80 min. Liquid water accumulation was observed at the top surface of the slab (Figure 22), and steam release was visible near the anchors and the thermocouple line. As a consequence, when considering the measured temperatures (instead of the theoretical temperatures), the predicted capacities at 30, 60 and 90 min became very similar. These predictions were very conservative at 30 min and 60 min, but less conservative at 90 min.

## 6. Conclusions

This study investigated the tensile concrete cone capacity of anchors in concrete subjected to elevated temperatures through an extensive experimental program combined with comparisons against existing and proposed design approaches. The experimental results showed that neither the concrete strength class (C20/25 versus C50/60) nor the presence of a crack had a significant influence on the anchor capacity for the tested conditions. For anchor groups, the overall capacity was governed by the most thermally affected anchor. Concrete screws showed a greater reduction in capacity than headed anchors, but the observed scatter did not allow for a definitive conclusion regarding the influence of anchor technology on the reduction. Nevertheless, the experimental results for screws were still significantly greater than those for the proposed calculation model. When comparing experimental results with calculated predictions, significant differences were observed even at ambient temperature, with calculated capacities less than measured values by up to 20%. Under elevated temperature conditions induced by radiant panel heating, the theoretical models were found to be highly conservative, in some cases underestimating the experimental results by more than 50%. For bottom gas-heating tests, the level of conservatism depended on the fire exposure duration, with predictions being either conservative or unconservative depending on the scenario considered. A direct comparison with EN 1992-4 is not straightforward, as the temperature fields obtained with radiant panels are not equivalent with those associated with ISO 834-1 fire exposure at 90 or 120 min. Nevertheless, irrespective of the temperature fields, the experimentally observed capacity reductions for exposure from the top (with electric heating) typically ranged between 10% and 50% for an embedment depth of h_ef_ = 100 mm. These reductions are generally lower than those prescribed by EN 1992-4 for the same embedment depth, which assumes a reduction of approximately 50%, indicating that the existing EN 1992-4 provisions are overly conservative for the results in this study. Section 4.2 shows that for embedment depths of 170 mm, the proposed design method yields capacities equal or higher than those from EN 1992-4, thus demonstrating optimized design in comparison to EN 1992-4, noting that this comparison with the EN 1992-4 method is only applicable for one-sided exposure for ratings at 90 min and 120 min. The main benefit is that the method allows designers to account for different fire exposure scenarios and different fire durations.

Overall, the findings suggest that the proposed design approach provides a safe estimation of tensile concrete cone capacity under fire conditions and may be overly conservative for certain exposure configurations. The method can be formulated as a temperature-based design procedure in three steps:

(1) Calculate the temperature at h_ef_ (the effective embedment depth) of the anchor at a given time during fire exposure.
(2) Determine the reduction factor k_c_ using Equation (9).
(3) Calculate the load capacity $N_{Rk,c}$.

The third step relies on two conclusions from the parametric analysis:

1. Under fire there is no additional reduction than the one already applied at 20 °C (0.7 factor) for cracked concrete. Thus, the multiplication factor under fire can be applied directly to the ambient cracked concrete capacity.

For anchor groups or anchors close to an edge, the reduction factor is applied to the reference value $\frac{A_{c,N}}{A_{c,N}^{0}}\cdot N_{Rk,c}^{0}$, where A_c,N_ and A^0^_c,N_ are calculated using the same parameters as ambient temperature (cone dimension based on 3.h_ef_). In the case of anchor groups, the temperature considered in the second step corresponds to that of the most critical anchor, i.e., the one experiencing the highest temperature at the embedment depth.

The test results do not indicate that the current EN 1992-4 method is unsafe. The proposed temperature-based method provides similar capacities to the existing design predictions of EN 1992-4, and it offers greater flexibility by accommodating different heating orientations and allowing capacity predictions for a broader range of fire exposure durations. Furthermore, the method enables optimized design by explicitly accounting for parameters influencing the temperature at the embedment depth of the anchor, including geometric characteristics and the presence of thermal protection. To fully benefit from this optimized design, temperature-based design formulations should be extended to other failure modes, such as steel, pullout, and splitting. Further investigations are also necessary to assess the applicability of this method for other loading directions such as shear loading under fire conditions.

## Figures and Tables

**Table 1:** Test program with experimental variables of interest shown in blue.

Line	Test Name	Nb. of Tests	Concrete	Concrete Strength	Single/Group	Embedment Depth	Anchor Technology	Heating Type	Fire Exposure
1	Reference 20 °C	5	Uncracked	C20/25	Single	h_ef_ = 100 mm	Headed anchor	20 °C	
2	Reference hot	5	Uncracked	C20/25	Single	h_ef_ = 100 mm	Headed anchor	Electric	Top
3	Reference cracked	3	Cracked	C20/25	Single	h_ef_ = 77 mm	HDA-P	20 °C	
4	Cracked	3	Cracked	C20/25	Single	h_ef_ = 77 mm	HDA-P	Electric	Top
5	Lateral heating	2	Uncracked	C20/25	Single	h_ef_ = 100 mm	Headed anchor	Electric	Lateral
6	Ref. close to edge	2	Uncracked	C20/25	Single	h_ef_ = 100 mm	Headed anchor	20 °C	
7	Two-sided heating	2	Uncracked	C20/25	Single	h_ef_ = 100 mm	Headed anchor	Electric	Top & lateral
8	Reference group	3	Uncracked	C20/25	Group × 2	h_ef_ = 100 mm	Headed anchor	20 °C	
9	Group hot	3	Uncracked	C20/25	Group × 2	h_ef_ = 100 mm	Headed anchor	Electric	Top
10	Group lateral	2	Uncracked	C20/25	Group × 2	h_ef_ = 100 mm	Headed anchor	Electric	Lateral
11	Bottom gas heating	5	Uncracked	C20/25	Single	h_ef_ = 92 mm	HDA-P	Gas	Bottom
12	Ref. C50/60 concrete	5	Uncracked	C50/60	Single	h_ef_ = 57 mm	HDA-P	20 °C	
13	C50/60 concrete	5	Uncracked	C50/60	Single	h_ef_ = 57 mm	HDA-P	Electric	Top
14	Ref. concrete screw	5	Uncracked	C20/25	Single	h_ef_ = 100 mm	HUS4	20 °C	
15	Concrete screw	5	Uncracked	C20/25	Single	h_ef_ = 100 mm	HUS4	Electric	Top

**Table 2:** Measured and predicted capacities.

Test	Test 1	Test 2	Test 3	Test 4	Test 5	Average Measured Capacity (kN)	Temperature at Deepest Part of the Embedment Depth (°C)	Theoretical Capacity (kN)
Reference Tests (headed bolt, h_ef_ = 100 mm, f_c_ = 34.4 MPa, uncracked)								
20 °C	97.8	91.9	85.6	79.9	87.4	88.5	20	88.6
Top heating	71	72.9	80.1	88.2	82.1	78.9	82–160	36.1–55.3
Edge Tests (headed bolt, h_ef_ = 100 mm, f_c_ = 34.4 MPa, uncracked, c_1_ = 100 mm)								
20 °C	57.7	57.1				57.4	20	73.8
Lateral heating	44.0	36.6				40.3	155	33.2
Top + lateral heating	33.0	32.7				32.9	375	14.8
Group Tests (headed bolt, h_ef_ = 100 mm, f_c_ = 34.4 MPa, uncracked, c_1_ = 100 mm, s = 150 mm)								
20 °C	133.1	133.8	129.5			132.1	20	118.1
Top heating	84.9	103.9	98.5			95.8	121	62.5
Lateral heating	101.9	85.5				93.7	127	60.7
Cracked Tests (HDA-P, h_ef_ = 77 mm, f_c_ = 34.4 MPa, cracked)								
20 °C	60.5	50.1				55.3	20	41.9
Top heating	62.4	51.3				56.9	118	22.5
C50/60 Tests (HDA-P, h_ef_ = 57 mm, f_c_ = 63.0 MPa, uncracked)								
20 °C	61.7	68.8	65.3			65.3	20	51.6
Top heating	27.4	28.0	34.8	39.0	32.3	32.3	220	17.7
Concrete Screw Tests (HUS4, h_ef_ = 100 mm, f_c_ = 33.0 MPa, uncracked)								
20 °C	57.4	58.8	53.9			56.7	20	54.5
Top heating	40.2	35.9	39.9	42.1	32.6	38.1	121	28.9

**Table 3:** Failure loads measured during the bottom-heating fire tests.

Time (min)	Failure Load (kN)	Calculated Temperature (°C)	Measured Temperature (°C)
0	55.0	20	20
30	47.1	26	100
60	42.7	57	102
90	18.2	92	110
90	24.8	129	110
120	22.1	129	190

## Data Availability

The original contributions presented in this study are included in the article. Further inquiries can be directed to the corresponding author.
